# Astragalus Extract Mixture HT042 Increases Longitudinal Bone Growth Rate by Upregulating Circulatory IGF-1 in Rats

**DOI:** 10.1155/2017/6935802

**Published:** 2017-06-20

**Authors:** Donghun Lee, Sung Hyun Lee, Yoon Hee Lee, Jungbin Song, Hocheol Kim

**Affiliations:** ^1^Department of Herbal Pharmacology, Kyung Hee University College of Korean Medicine, Seoul 02447, Republic of Korea; ^2^Korea Institute of Science and Technology for Eastern Medicine (KISTEM), NeuMed Inc., Seoul 02440, Republic of Korea

## Abstract

Astragalus extract mixture HT042 is a standardized ingredient of health functional food approved by Korean FDA with a claim of “height growth of children.” HT042 stimulates bone growth rate and increases local IGF-1 expression in growth plate of rats which can be considered as direct stimulation of GH and its paracrine/autocrine actions. However, it remains unclear whether HT042 stimulates circulatory IGF-1 which also plays a major role to stimulate bone growth. To determine the effects on circulatory IGF-1, IGF-1 and IGFBP-3 expressions and phosphorylation of JAK2/STAT5 were evaluated in the liver after 10 days of HT042 administration. HT042 upregulated liver IGF-1 and IGFBP-3 mRNA expression, IGF-1 protein expression, and phosphorylation of JAK2/STAT5. HT042 also increased bone growth rate and proliferative zonal height in growth plate. In conclusion, HT042 stimulates bone growth rate via increment of proliferative rate by upregulation of liver IGF-1 and IGFBP-3 mRNA followed by IGF-1 protein expression through phosphorylation of JAK2/STAT5, which can be regarded as normal functioning of GH-dependent endocrine pathway.

## 1. Introduction

Longitudinal bone growth is the result of chondrocyte proliferation and ensuing hypertrophy in growth plates, called endochondral ossification which is mainly controlled by a system of growth hormone- (GH-) insulin-like growth factor-1 (IGF-1) axis [1]. Mammalian growth plate consists of three principal zones: resting, proliferative, and hypertrophic zones. In resting zone, GH begins its actions by direct stimulation of resting stem-like chondrocytes to start proliferation. The following cell replication in proliferative zone and terminal differentiation and enlargement in hypertrophic zone are both primarily caused by circulatory IGF-1 [2, 3] and locally expressed IGF-1 [4].

Circulatory IGF-1 is biosynthesized in the liver as an endocrine hormone in the ternary form bound to IGF binding protein-3 (IGFBP-3) and acid labile subunit (ALS), while IGF-1 is also expressed by nonliver tissues including growth plate, where it works in a paracrine/autocrine fashion [5]. For better understanding of the endocrine versus the paracrine/autocrine roles of IGF-1 in bone growth, previous studies have been used to examine the relative contribution to longitudinal bone growth. It has been widely reported that longitudinal growth is mainly mediated by paracrine/autocrine actions of IGF-1 based on the Yakar et al. study that liver IGF-1 deficient mice developed normally in spite a 75% reduction in serum IGF-1 levels due to compensatory serum GH and nonhepatic IGF-1 [6, 7]. But, years later, the same team revealed that circulatory IGF-1 is important for maintaining normal growth by the study that liver IGF-1 and ALS double knockout mice were much smaller because there was a decrease in not only IGF-1 production but also circulatory IGF-1 stability that originated from compensatory nonliver sources because of the absence of protective ALS protein [3]. This suggests that circulatory IGF-1 plays a major role in longitudinal bone growth rate.

Almost 80% of circulatory IGF-1 is produced by liver in humans and rodents. It is well established that circulatory IGF-1 levels are regulated posttranscriptionally mainly by GH at the postnatal period [8]. For production of circulatory IGF-1, actions of GH are started by its binding to GH receptor (GHR) on the surface of liver cells [9]. This causes dimerization of receptor and phosphorylation of GHR-related tyrosine kinase Janus kinase 2 (JAK2) [10]. Phosphorylated JAK2 phosphorylates signal transducers and activators of transcription 5 (STAT5) and phosphorylated STAT5 translocate to the nucleus and stimulates the IGF-1 transcription [11].

Astragalus extract mixture, HT042, consisting of the roots of* Astragalus membranaceus* and* Phlomis umbrosa *and the stem of* Eleutherococcus senticosus*, is a standardized ingredient of health functional foods approved by Korean FDA with a claim that “HT042 may help height growth of children” based on the evidence derived from randomized controlled trials. HT042 stimulates longitudinal bone growth rate in rats and increases the expressions of local IGF-1 in the proliferative and hypertrophic zones of the tibial growth plate which can be considered as direct stimulation of GH and its paracrine/autocrine actions [12–14]. However, it remains unclear whether HT042 stimulates circulatory IGF-1 which plays a major role to stimulate longitudinal bone growth.

To evaluate whether HT042 affects circulating IGF-1, we evaluated liver IGF-1 and IGFBP-3 mRNA expressions by quantitative real-time PCR and liver IGF-1 protein levels and the phosphorylation of JAK2/STAT5 by western blot in response to HT042 administration. We observed that HT042 increased bone growth rate and zonal height of growth plate of proximal tibia. Tetracycline was employed as a fluorescent reagent to identify anew formed bone by the growth plate.

## 2. Methods

### 2.1. Sample

Standardized HT042 was provided from NeuMed (Korea). Manufacturing process and quality management of HT042 were conducted in accordance with the methods registered in Korean FDA. Standardized HT042 contains shanzhiside methyl ester 0.15%, eleutheroside E 0.36%, and formononetin 0.008%. For the quality verification, the quantitative analysis of HT042 was performed by high-performance liquid chromatography (HPLC) for measuring concentrations of marker compounds of* A. membranaceus*,* E. senticosus,* and* P. umbrosa *(formononetin, eleutheroside E, and shanzhiside methyl ester, resp.).

### 2.2. Animals

Female Sprague-Dawley rats with an age of 25 days were bought from Samtako (Korea). The present study was performed according the guidelines of the Institutional Animal Care and Use Committee, Korea Institute of Science and Technology for Eastern Medicine (KISTEM-IACUC-2016-001). Rats were housed under constant temperature and humidity conditions with food and water ad libitum.

### 2.3. Treatment

After 7-day acclimatization, rats were assigned into 3 groups: vehicle, recombinant human GH (rhGH, Eutropin, LG, Korea) 200 *μ*g/kg, and HT042 100 mg/kg. HT042 or distilled water vehicle was orally treated twice daily (8:00 am; 8:00 pm) and rhGH was subcutaneously treated once daily (8:00 am) for 10 days.

### 2.4. Real-Time Quantitative Polymerase Chain Reaction (PCR) Analysis

After sacrifice, livers were promptly removed, rinsed, and stored at −80°C. Total RNA from liver samples was extracted using QIAzol reagent (Qiagen, USA) and converted into cDNA with transcription kit (Applied Biosystems, USA) according to the instructions. Quantitative PCR was performed on a real-time PCR system (Applied Biosystems, USA) as follows: after 10 m at 95°C, 40 cycles of 15 s at 95°C and 60 s at 60°C. Primers were designed and synthesized by Bioneer (Korea): insulin-like growth factor-1 (GenBank Accession Number M15481), forward 5-GCTATGGCTCCAGCATTCG-3 and reverse 5-TCCGGAAGCAACACTCATCC-3; insulin-like growth factor-binding protein-3 (GenBank Accession Number NM_012588), forward 5-GGAAAGACGACGTGCATTG-3 and reverse 5-GCGTATTTGAGCTCCACGTT-3; glyceraldehyde-3-phosphate dehydrogenase (GAPDH, GenBank Accession Number NM_017008), forward 5-TGGCCTCCAAGGAGTAAGAAAC-3 and reverse 5-CAGCAACTGAGGGCCTCTCT-3. Conditions were as follows: 10 min at 95°C and 40 cycles of 15 s at 95°C and 60 s at 60°C. Relative quantifications was performed using the delta-delta Ct method with normalization to GAPDH expression.

### 2.5. Growth Plate Height

Cresyl violet (CV, Sigma-Aldrich, USA) was used to stain chondrocytes for measuring height of growth plate. Overall growth plate height and zonal height were measured by using ImageJ software (NIH, USA) at 3 locations as previously described [15].

### 2.6. Measuring Longitudinal Bone Growth Rate

Tetracycline hydrochloride (20 mg/kg, i.p., Sigma-Aldrich) was injected 72 h before sacrifice as previously described [15]. Dissected tibias were fixed with 4% paraformaldehyde, decalcified in 50 mM EDTA solution (Sigma-Aldrich) and dehydrated in 30% sucrose solutions overnight. Dehydrated tibias were sectioned in the sagittal plane with 40 *μ*m thickness on a cryostat (CM3050S, Leica Microsystems, Germany). Daily longitudinal growth rate was estimated by dividing the distance between tetracycline label and chondroosseous junction into three. Tetracycline label was examined with a fluorescent microscope (BX50, Olympus, Japan) and the distance was measured with ImageJ by three different researchers in a blind manner. Rats for measuring longitudinal bone growth rate were used separately because tetracycline injection can affect other parameters.

### 2.7. Western Blotting

Protein from rat liver samples was extracted using the PRO-PREP™ (iNtRON Biotechnology, Korea) in accordance with the manufacturer's instructions. Total protein concentrations were quantified by Bradford reagent (Bio-Rad, USA). Total protein 50 *μ*g was denatured in load buffer and separated using SDS-PAGE on Bolt™ 4–12% Bis-Tris gel (Invitrogen, USA). After electrophoresis, separated proteins were electrotransferred onto PVDF membranes (Merck Millipore, Germany). The membrane was blocked with 5% skim milk for 1 h at room temperature. After the blocking, membranes were incubated overnight at 4°C with the respective primary antibodies: mouse monoclonal anti-*β*-actin antibody (Sigma-Aldrich, USA; diluted 1 : 20000), mouse monoclonal anti-IGF-1 antibody (Abcam; diluted 1 : 2500), rabbit polyclonal anti-STAT5 antibody (Cell Signaling Technology; diluted 1 : 1000), rabbit polyclonal anti-phospho-STAT5 antibody (Tyr694, Cell Signaling Technology; diluted 1 : 1000), rabbit polyclonal anti-JAK2 antibody (Cell Signaling Technology; diluted 1 : 1000), and rabbit polyclonal anti-phospho-JAK2 antibody (Tyr1008, Cell Signaling Technology; diluted 1 : 1000). After washing, the membrane was incubated with horseradish peroxidase-conjugated secondary anti-mouse IgG antibody (Sigma-Aldrich, USA; diluted 1 : 3000) or horseradish peroxidase-conjugated secondary anti-rabbit IgG antibody (Sigma-Aldrich, USA; diluted 1 : 3000). The membranes were developed by using ECL prime detection reagent (GE Healthcare, UK). Protein expression was detected with an Amersham Imager 600 (GE Healthcare, UK).

### 2.8. Enzyme-Linked Immunosorbent Assay (ELISA)

For the quantitative analysis of the serum IGF-1 concentration, a sandwich assay for IGF-1 was carried out in duplicate in a 96-well ELISA kit according to the manufacturer's protocol (RMEE25R, BioVendor, Czech Republic).

### 2.9. Statistical Analysis

Statistical analysis was performed with GraphPad Prism 6 software (USA). One-way analysis of variance with post hoc Newman-Keuls test was used for multiple comparisons. Statistical significance was set at *p* < 0.05. All values were presented as mean ± SD.

## 3. Results

### 3.1. Quantitative Analysis of HT042

**Figure 1:**
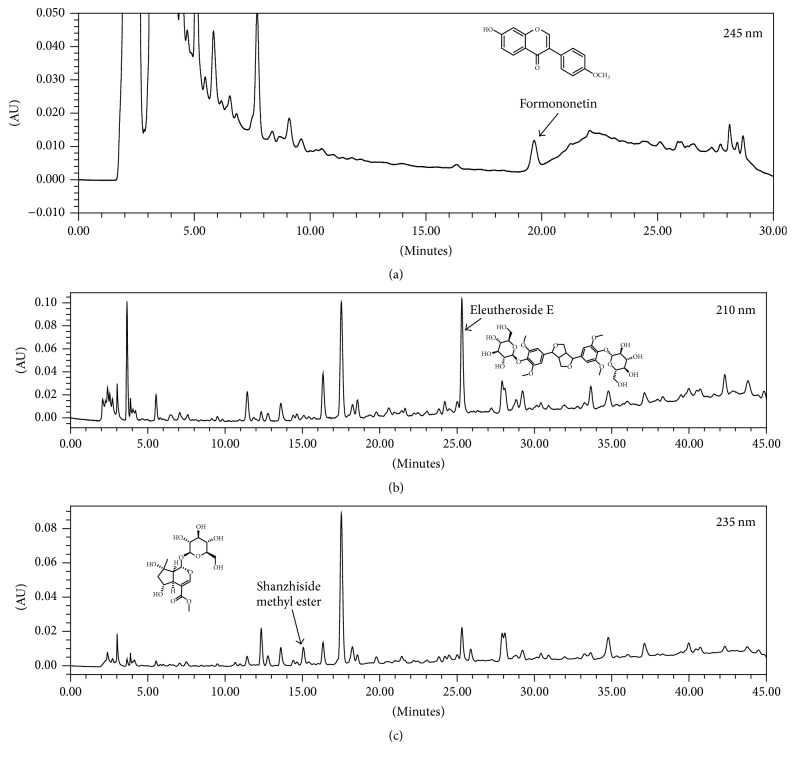
HPLC chromatograms of (a) formononetin, (b) eleutheroside E, and (c) shanzhiside methyl ester, the marker compounds of* A. membranaceus*,* E. senticosus*, and* P. umbrosa,* respectively, in the HT042.

Quantitative analysis of HT042 by HPLC was performed to determine the concentrations of the marker compounds for quality assurance. The result showed that the concentrations of formononetin, eleutheroside E, and shanzhiside methyl ester were in the range of 64.0–96.0 *μ*g/g, 2.88–4.32 mg/g, and 1.20–1.80 mg/g, respectively (Figure 1).

### 3.2. Effect on IGF-1 and IGFBP-3 mRNA Expression in Liver

**Figure 2:**
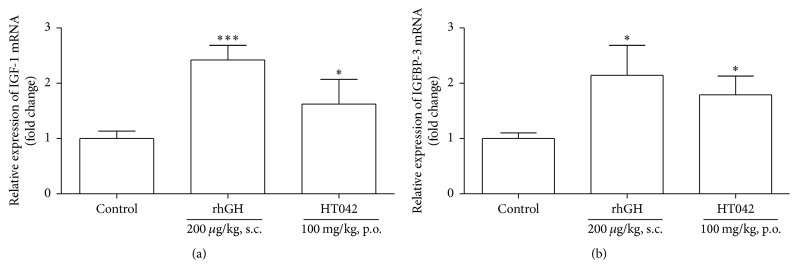
The relative expression of IGF-1 and IGFBP-3 mRNA in the liver measured by quantitative real-time PCR. Relative transcriptional levels of (a) IGF-1 and (b) IGFBP-3 were analyzed by applying to GAPDH internal control; ^*∗*^*p* < 0.05 and ^*∗∗∗*^*p* < 0.001 versus control.

Previously, we showed that HT042 administration increased IGF-1 expression on the local growth plate of normal rats [12–14]. To evaluate whether HT042 affects circulating IGF-1, quantitative real-time PCR was conducted to measure liver IGF-1 and IGFBP-3 mRNA expressions because serum IGF-1 is synthesized primarily in the liver in a GH-dependent manner [16]. Oral administration of HT042 100 mg/kg significantly increased mRNA expression of IGF-1 and IGFBP-3 in the liver exhibiting 1.6- and 1.4-fold values compared to the control group, respectively, although the increment was smaller compared to rhGH (Figure 2).

### 3.3. Effect on IGF-1 Protein Expression and JAK2/STAT5 Phosphorylation in the Liver

**Figure 3:**
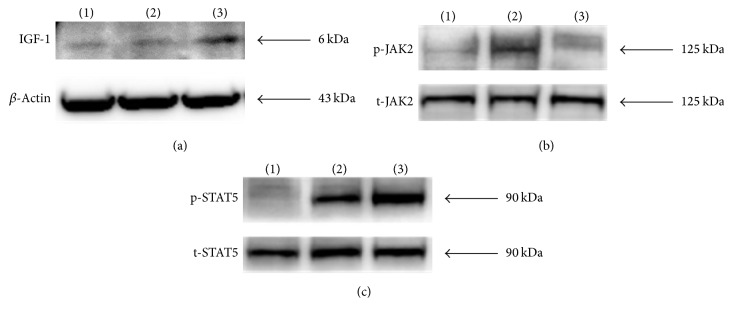
The protein levels of IGF-1 and JAK2/STAT5 in the liver measured by western blot. (a) IGF-1, (b) phosphor- (p-)JAK2/total- (t-)JAK2, and (c) p-STAT5/t-STAT5; (1) control group, (2) rhGH 200 *μ*g/kg (s.c.) treated group, and (3) HT042 100 mg/kg (p.o.) treated groups.

To determine whether growth-stimulating effect of HT042 was mediated by the phosphorylation of JAK2 and STAT5 which cause IGF-1 production, we performed western blotting. As expected, IGF-1 protein was predominantly expressed in HT042 group compared to control group. Phosphorylations of JAK2 and STAT5 in the liver were upregulated by HT042 (Figure 3).

### 3.4. Effect on Circulating IGF-1 Level

**Figure 4:**
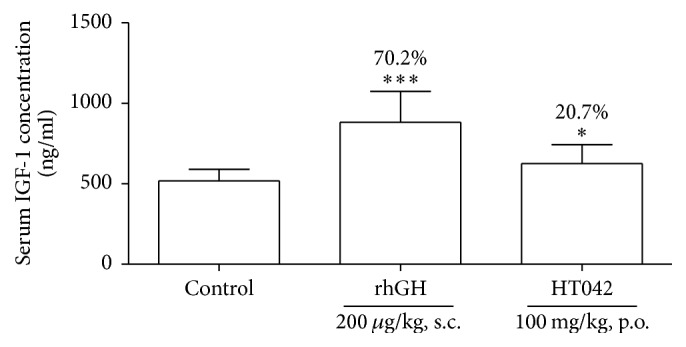
The serum concentration of IGF-1 measured by ELISA; ^*∗*^*p* < 0.05 and ^*∗∗∗*^*p* < 0.001 versus control.

To examine whether HT042 increases circulating IGF-1 level, we measured the serum IGF-1 concentration by ELISA.  Figure 4 shows the numerical value of serum IGF-1 concentration of each group. Serum IGF-1 concentration in the control group was 518.4 ± 72.2 ng/mL. Administration of rhGH and HT042 for 10 days significantly increased the serum IGF-1 exhibiting 882.1 ± 192.5 ng/mL and 625.8 ± 117.9 ng/mL compared to the control group, respectively.

### 3.5. Effect on Longitudinal Bone Growth Rate

**Figure 5:**
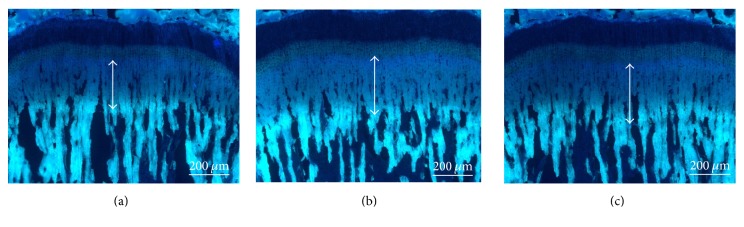
Typical fluorescent photography of proximal growth plate of tibia. The double arrows indicate the distance of bone growth in proximal tibia for 72 h. (a) Control; (b) rhGH 200 *μ*g/kg (s.c.); (c) HT042 100 mg/kg (p.o.). Scale bar, 200 *μ*m.

**Figure 6:**
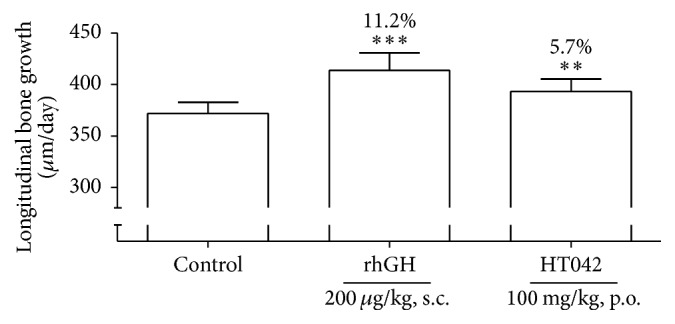
Effects of HT042 on bone growth rate in proximal growth plate of tibia; ^*∗∗*^*p* < 0.01 and ^*∗∗∗*^*p* < 0.001 versus control.

To assess the effect of HT042 on bone growth rate, tetracycline was employed as a fluorescent reagent to mark freshly formed bone beneath growth plate. The double arrows indicate the distance of bone growth in proximal tibia for 72 h (Figure 5).  Figure 6 shows the numerical value of bone growth rate of each group. Bone growth rate in the control group was 378.5 ± 24.6 *μ*m/day. Administration of rhGH and HT042 significantly increased the bone growth rate exhibiting 406.9 ± 24.1 *μ*m/day and 393.2 ± 18.8 *μ*m/day compared to the control group, respectively.

### 3.6. Effect on the Growth Plate Height

**Figure 7:**
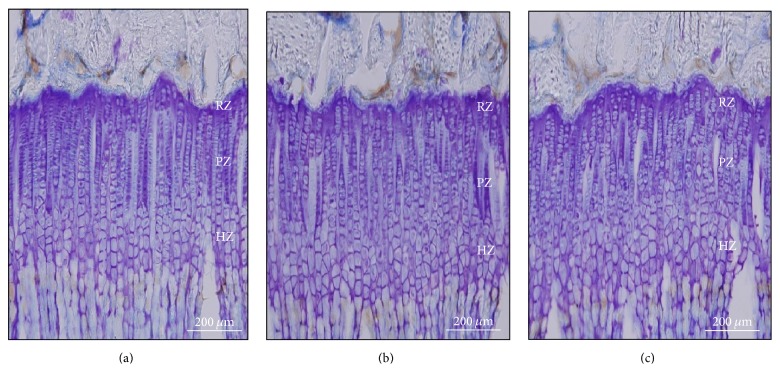
Photography of cresyl violet-stained chondrocytes in proximal growth plate of tibia; (a) control, (b) rhGH 200 *μ*g/kg (s.c.), and (c) HT042 100 mg/kg (p.o.). Scale bar, 200 *μ*m.

Height of proximal tibial growth plate was measured with CV staining (Figure 7). Overall growth plate height was 350.3 ± 19.6 *μ*m in control group. Administration of rhGH and HT042 significantly increased height of growth plate exhibiting 365.4 ± 17.3 *μ*m and 365.6 ± 23.9 *μ*m compared to the control group, respectively. Height of proliferative zone, particularly, was significantly increased in HT042 group compared to control (Table 1).

## 4. Discussion

Oral administration of Astragalus extract mixture HT042 at a dose of 100 mg/kg for 10 days upregulated liver IGF-1 mRNA and protein expression and increased circulating IGF-1 level via the phosphorylation of JAK2/STAT5. HT042 also significantly increased height of proliferative zone and longitudinal bone growth rate and increased the local IGF-1 and BMP-2 expressions in growth plate.

HT042 showed significant upregulation of liver IGF-1 and IGFBP-3 mRNA expression compared to control. Liver is the main source of circulatory IGF-1 and IGFBP-3 in a manner dependent on GH [17]. In a mammalian, almost 80% of circulatory IGF-1 is carried by IGFBP-3 to prolong the half-life of IGF-1 and transport IGF-1 to its receptor [18] and diminishing of IGFBP-3 weakens its IGF-1 protective ability. For these reasons, serum levels of IGF-1 and IGFBP-3 are thought to be excellent biochemical parameters reflecting GH level which is widely fluctuating over time [19, 20]. The positive impact of the HT042 on IGF-1 or IGFBP-3 mRNA expression is in accordance with previous studies reporting the increasing effect of formononetin and *β*-glucan of* A. membranaceus*,* A. senticosus* polysaccharide, and* P. umbrosa* extracts on serum IGF-1 or IGFBP-3 concentration [15, 21, 22]. In the previous study, we found that HT042 does not alter bone growth rate of spontaneous dwarf rat which can not produce intact GH mRNA, suggesting HT042 may stimulate GH secretion rather than act like GH [12, 23]. Taken together, the results suggest that the effects of HT042 on bone growth rate might be attributable to the increment of circulatory IGF-1 and IGFBP-3 by the stimulation of GH.

HT042 increased circulating IGF-1 level and liver IGF-1 protein expression with the increment of phosphorylation of JAK2/STAT5 protein. It is well established that circulatory IGF-1 is mainly produced in the liver and regulated posttranscriptionally by GH at the postnatal period. GH signaling is mediated by protein phosphorylation cascades, thereby activating nuclear proteins and transcription factors [24]. Binding of GH to its receptor leads to activation of JAK2, which in turn phosphorylates STAT5. Then, STAT5 translocates to the nucleus, binds to certain DNA sequence, and regulates transcription [25]. Among the signaling cascades induced by GH, JAK2/STAT5 signaling cascade plays a major role in GH regulation of gene transcription in liver [26]. Moreover, this pathway has been reported to be in charge of GH-induced liver IGF-1 expression [27]. Our results showing that HT042 increases IGF-1 levels in both serum and liver with the phosphorylation of JAK2/STAT5 suggest that the growth-stimulating effect of HT042 is mediated by the increment of circulating IGF-1 through the JAK2/STAT5 signal cascade.

When liver IGF-1 production is increased, circulating level of ternary complexes consisting of IGF-1, IGFBP-3, and ALS is increased in serum and then isolated IGF-1 binds to IGF-1 receptors of local growth plates and stimulates proliferation of chondrocytes which cause increment of the height of growth plates [3]. To assess this, we analyzed the zonal height of proximal growth plate in rat tibia. HT042 significantly increased overall growth plate height by 4.4% particularly proliferative zonal height by 11.4% compared to control. Among three characteristic areas of growth plate, fast division at proliferative zone and ensuing expansion at hypertrophic zone result in bone growth rate and height of growth plate. Particularly, the greatest contribution to bone growth rate is the rate of clonal proliferation which results in increase in proliferative zone height [5]. Previous researchers have detected close correlations between proliferative zonal height and bone growth rate regardless of other factors [28–30]. The result suggests that HT042 increases proliferation rate of the chondrocytes in growth plate by upregulation of liver IGF-1 and IGFBP-3 expressions.

We already reported that HT042 at doses of 100 mg/kg for 4 consecutive days increased the bone growth rate measured by tetracycline in the proximal tibial growth plate. This method is hard to evaluate the efficacy accurately because of the excessive individual difference of rats and researchers. In the present study, rats with exactly the same days of age were used because the bone growth rate of rats varies excessively depending on the days of age. The distances were blind-read by three different researchers to avoid the distinction among individuals. Administration periods were extended to 10 consecutive days from 32-day-old to 41-day-old rats because rats reach sexual maturity at approximately 50 days of age and in the adolescent period 10 rat days are converted into 1 human year [31]. Oral administration of HT042 at a dose of 100 mg/kg for 10 days showed the significant increase of the longitudinal bone growth rate compared to the control group under well controlled condition in accordance with previous result suggesting that HT042 increases longitudinal bone growth rate.

## 5. Conclusion

Astragalus extract mixture HT042 stimulates bone growth rate via upregulation of liver IGF-1 and IGFBP-3 mRNA followed by IGF-1 protein levels in serum and liver through the phosphorylation of JAK2/STAT5, which can be considered as normal functioning of GH-dependent endocrine pathway.

## Figures and Tables

**Table 1:** Height of growth plate in each group.

*μ*m	Control	rhGH 200 *μ*g/kg (s.c.)	HT042 100 mg/kg (p.o.)
Overall height	350.3 ± 19.6	365.4 ± 17.3^*∗*^	365.6 ± 23.9^*∗*^
Resting zone	22.0 ± 5.3	21.4 ± 5.2	20.2 ± 3.8
Proliferative zone	119.9 ± 11.5	127.3 ± 15.7	133.6 ± 11.2^*∗∗*^
Hypertrophic zone	198.5 ± 12.7	203.3 ± 17.6	205.3 ± 20.8

^*∗*^ *p* < 0.05 and ^*∗∗*^*p* < 0.01 versus control.
